# The "Infamy" of Advertising

**Published:** 1901-06-15

**Authors:** 


### The "Infamy" of Advertising.

At the meeting of the General Medical Council held last Saturday the charge against Dr. Irvine was again considered, and the case terminated as we last week suggested it probably would do, the President informing Dr. Irvine that the Council, "having considered the facts of the case and the evidence submitted as to his action since the last session, had resolved to proceed no further in regard to the charge proved against him." The profession will thank Sir William Gairdner for not allowing the case to come to an end without obtaining a distinct admission from Dr. Irvine that he quite understood all the time that the *gravamen* of the charge against him was the advertisements and not the reduced fees. In view of the obstinacy with which people occupying responsible positions, even in Parliament, have maintained the ignorant view that the whole trouble has been a mere trades union fight to keep up the fees of the Birmingham consultants, it is important that this admission should have been put on record.

So far as Dr. Irvine is concerned, we may presume that the incident is now closed. It is impossible, however, to cover over the fact that the General Medical Council has by no means enhanced the dignity of its position by the way in which it has conducted and ultimately got out of this affair. It is perhaps to be regretted that by the way this case has terminated we are deprived of a pronouncement, which would certainly have proved interesting, and perhaps useful to not a few, as to the degree and kind of advertising which the Council considers infamous, and is prepared to punish by expulsion from the Register. It is to be noted that the charge brought against Dr. Irvine was that he "approved or acquiesced in" the advertising which had taken place, and that as the result of their deliberations the Council decided that "the charge which he had been required to answer had been proved." But the Council by no means asserted in their charge, and were careful to avoid asserting in their judgment, the only thing which in fact would have given them any jurisdiction—namely, that the particular conduct proved against him was, in fact, "infamous in a professional respect." Except in cases in which a registered practitioner has been guilty of felony or misdemeanour, the only charge upon which the General Medical Council can act is one of "infamous conduct in any professional respect," and unless they are prepared to say, not merely, as they have said, that the charges are proved, but also, as they have not said, that what was done was "infamous," they have no jurisdiction.

No doubt the Council thought they had a rod in pickle for Dr. Irvine, and no doubt the fear of being turned off the Register is a very wholesome stimulus to rectitude among some of our shaky brethren; but if this case is to have a moral we ought to know what really is considered infamous in the way of advertising. At present medical men are in the predicament of not being able to ascertain how far they may go in a practice which is unfortunately and admittedly far too common. Granted that advertising is a sin of many shades—so many that in some forms it seems to be accepted almost as a virtue—and granted that, in vulgar phrase, it is always difficult to know "where lamb ends and mutton begins," still the General Medical Council is a judicial body, and to the medical man who has sunk his all in getting on the Register its thunders are very terrible; and we think that it would have been very satisfactory, even at the cost of sacrificing half a dozen Dr. Irvines, to have obtained a distinct pronouncement as to what sort of advertising is so "infamous" as to involve removal from the Register. Between an ordinary door-plate and such advertisements as those issued by the Birmingham Consultative Institution there are many grades, and somewhere in between these two extremes come a whole crowd of medical books, some of which we fear are written with the very object of showing the writers' "special ability," to quote the words of the charge against Dr. Irvine, and we should very much like to see the Council attempt to lay down some sort of principle and draw some sort of line! In the meantime, and until it can do this, is it not a little undignified to hurl thunderbolts which, for aught we know, might be pricked as bubbles by the Privy Council or in the law courts?

